# Tumors of the labial mucosa: a retrospective study of 1045 biopsies

**DOI:** 10.4317/medoral.23933

**Published:** 2020-08-27

**Authors:** Eleni Marina Kalogirou, Maria G. Balta, Marianna Koufatzidou, Athina Tosiou, Konstantinos I. Tosios, Nikolaos G. Nikitakis

**Affiliations:** 1 DDS, MSc, PhD Candidate, Department of Oral Medicine and Pathology, Faculty of Dentistry, National and Kapodistrian University of Athens, Athens, Greece; 2 DDS, MSc, PhD Candidate, Institute of Oral Biology, University of Oslo, Oslo, Norway; 3 DDS, Private Practice, Athens, Greece; 4 Undergraduate Student, Faculty of Dentistry, National and Kapodistrian University of Athens, Athens, Greece; 5 DDS, PhD, Associate Professor, Department of Oral Medicine and Pathology, Faculty of Dentistry, National and Kapodistrian University of Athens, Athens, Greece; 6 MD, DDS, PhD, Professor and Head, Department of Oral Medicine and Pathology, Faculty of Dentistry, National and Kapodistrian University of Athens, Athens, Greece

## Abstract

**Background:**

To investigate the relative frequency of localized mucosal swellings of the upper and lower labial mucosa, the clinical-pathological diagnosis agreement and whether patient’s age and gender and tumor’s site and size may raise the suspicion of neoplasm.

**Material and Methods:**

Retrospective analysis was performed on upper or lower labial mucosal tumors, histopathologically diagnosed between 2009-2018. The diagnostic categories developmental/reactive tumors, benign and malignant neoplasms were associated with patient’s age and gender and tumor’s site and size; clinical-pathological diagnosis agreement was, also, evaluated.

**Results:**

Overall, 1000 (95.7%) developmental/reactive tumors, 35 (3.3%) benign and 10 (1%) malignant neoplasms were found. Upper/lower lip tumor ratio was 0.14:1. The diagnostic category was significantly associated with age (*p*<0.0001), site (*p*<0.0001) and diameter (*p*<0.0001). Age ≥60 years, tumor’s location on the upper lip and diameter >1cm were independent predictors for neoplasms. Patients presenting 2 or 3 of these variables were 20.2 times (*p* < 0.0001) or 33.6 times (*p* <0.0001), respectively, more likely to have a neoplasm. Complete/partial agreement between clinical and pathological diagnosis was seen in 96.3% of the cases.

**Conclusions:**

Most lip tumors involve the lower lip and are reactive, but upper lip tumors measuring >1cm in patients≥60 years have significantly higher probability to be neoplasms.

** Key words:**Lip, tumor, neoplasm, carcinoma, cyst.

## Introduction

Lips have a prominent position in the human face and therefore any lip lesion will be identified early and usually prompt the patient to ask for medical advice, either due to diagnostic or aesthetic purposes (1,2). Lips are prone to trauma, due to their mobility and contact with the teeth, and are also susceptible to the harmful effect of numerous extrinsic factors, such as foods, tobacco or ultraviolet radiation, while some of them may even be carcinogenic (3,4).

Large population-based surveys have shown that lip lesions, including tumors, are commonly encountered in clinical practice (5,6). Epidemiological information may be helpful for constructing the differential diagnosis list that will dictate further diagnostic work-up or management. However, only a few studies have investigated the epidemiology of lip lesions (3,7-10), but include both cutaneous and mucosal lesions, without a focus on tumors or localized mucosal enlargements.

Studies have shown that lesions of the lower lip are more frequent than those of the upper lip and are usually reactive (3,7,9). In our clinical experience, a localized mucosal swelling of the upper lip is more likely a neoplasm, compared with one in the lower lip. Therefore, the main aim of the present study was to evaluate this clinical observation by recording and comparing the relative frequency of localized mucosal swellings of the upper and lower labial mucosa. Moreover, we compared the clinician’s impression, reflected in the clinical diagnosis, with the histopathological diagnosis and investigated whether the age and gender of the patient and the site and size of the tumor might raise the suspicion of a neoplasm.

## Material and Methods

This is a retrospective study on biopsies accessioned in the Department of Oral Medicine and Pathology between the years 2009-2018. The preprinted biopsy request forms of lesions located on the upper or lower labial mucosa and marked by the submitting clinicians as “tumors” were collected. Tumors on the vermilion border or the skin of the lips were excluded from the study. Information on patients’ age and gender, site and maximum clinical size of the lesion, as reported by the submitting clinician, as well as clinical diagnosis were retrieved and stored in a Microsoft Office Excel 2010 spreadsheet. Gender (male or female) and site (upper lip or lower lip) were stored as categorical data with two subcategories, while age was stored as a numerical variable (exact age in years), in order to calculate the mean age, and a categorical variable (age in decades) with nine subcategories. Maximum diameter was initially stored as numerical data (maximum diameter in cm), but in order to minimize the underlying discrepancies in measurements of maximum diameter, numerical data were converted to categorical data. When both the incisional and excisional biopsies were available for the same case, only the information from the incisional biopsy request form was included in the analysis, as the clinician excising a previously biopsied lesion is aware of the diagnosis.

During the analysis, the tumors were tabulated in three diagnostic categories based on the final diagnosis: developmental/reactive, benign neoplasms and malignant neoplasms (11). We graded the agreement between the clinical and pathologic diagnosis as follows: grade 0, complete agreement, both diagnoses were identical; grade 1, partial agreement, different diagnoses, but of the same diagnostic category; grade 2, disagreement, different diagnoses, from different categories. When a differential diagnosis was present, the first in the list was defined as the clinical diagnosis. Cases with no clinical diagnosis were excluded from this part of the analysis.

Statistical analysis was performed with the SPSS, v25.0 Software for Windows (SPSS Inc., Chicago). Associations between categorical variables, e.g. gender, age in decades, site, lesion diameter (independent variables) and diagnostic group (dependent variable), as well as diagnostic group (independent variable) and grade of clinical-pathological diagnosis agreement (dependent variable), were evaluated by Chi-square test for pairwise comparisons. Fisher's exact test was used when expected frequency was <5. The mean age of the different diagnostic groups was compared by Kruskal Wallis test. Binary logistic regression model was used to assess a set of clinical and demographic parameters (lip tumor site, diameter above 1cm, age≥ 60 years and gender) as predictor variables for the presence of neoplasms. For all statistical evaluations, a two-tailed probability (p) value of <0.05 was considered as significant.

The study has been independently reviewed and approved by the Research Ethics Committee of the Faculty of Dentistry, National and Kapodistrian University of Athens, Greece (NKUA code number 420).

## Results

In the 10-year study period 10,897 biopsies were accessioned in our Department; 1407 (12.9%) of them were from the labial mucosa and 1045 (74.3%) of the latter were described as tumors. Excision had been performed in 1029 (98.5%) cases.

The present study included 128 (12.25%) upper lip tumors and 917 (87.75%) lower lip tumors, with an upper to lower lip tumors ratio of 0.14:1. In Table 1 the tumors of the upper and lower lip are tabulated according to the diagnostic group. Overall, 1000 (95.7%) developmental/reactive tumors, 35 (3.3%) benign and 10 (1%) malignant neoplasms were found. Most developmental/reactive tumors were mucous cysts (452, 45.2%), i.e. 439 mucous extravasation cysts (mucoceles) and 13 mucous retentions cysts.

**Table 1:**
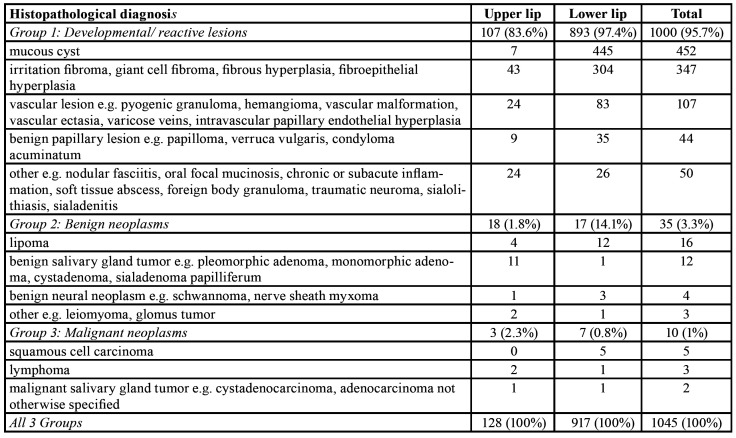
Site of 1045 localized mucosal swellings of the lips according to the diagnostic group.

Most mucous cysts of the lower lip were extravasation cysts (n=438, 99.8%) and of the upper lip were retention cysts (n=6/7), the difference being statistically significant (*p*<0.0001). Mucous extravasation cysts were found in younger patients (mean age 26.45±13.39 years) and mucous retention cysts in older patients (mean age 58.17±18.40 years), the difference being statistically significant (*p*<0.0001). Other common developmental/reactive tumors were fibromas (n=347, 34.7%) and vascular lesions (n=107, 10.7%). Most benign neoplasms were lipomas (n=16, 45.7%) or adenomas (n=12, 34.3%), including six cases of pleomorphic adenoma, and one each of canalicular adenoma, papillary cystadenoma and sialadenoma papilliferum. Malignant neoplasms included five squamous cell carcinomas, three lymphomas and two adenocarcinomas.

Table 2 summarizes the distribution of tumors in each diagnostic group, with respect to gender and age of the patients, upper/lower lip localization and maximum dimension. There were 598 female and 447 male patients (female to male ratio 1.34:1), without significant gender differences among the diagnostic groups. Age ranged between 3 and 89 years (mean age 40.68±19.87 years, data not available in 53 cases). The mean age of male (38.54±19.57 years) and female patients (42.28±19.96 years) did not differ significantly (*p*>0.05). A statistically significant relationship was noticed between patient’s age and the diagnostic group: most developmental/reactive tumors were diagnosed in patients between the 3rd and 5th decades of life (mean age 39.98±19.55 years), most benign neoplasms between the 7th and 8th decades of life (mean age 52.06±20.88 years), and most malignant tumors between the 7th and 9th decades of life (mean age: 70.0±13.91 years, *p*<0.0001). One hundred forty six tumors (13.97%) were diagnosed in children and adolescents and almost all of them (n=144, 98.63%) were developmental/reactive, with mucous cysts representing 83.33% of them.

**Figure 1:**
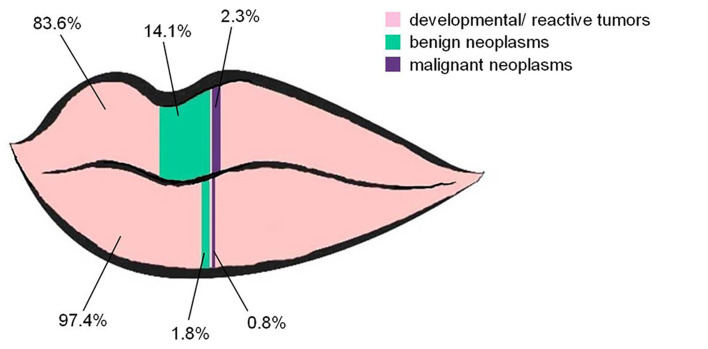
Relative frequency of 1045 upper and lower lip localized swellings of different diagnostic categories. Benign neoplasms are significantly more frequent among the upper than the lower lip tumors, corresponding to 14.1% and to 1.8% of lip tumors, respectively (Chi-square test, <italic>p</italic> <0.0001).

As it is shown in Table 2 and depicted in Fig. 1, the upper/lower lip location was significantly associated with the diagnostic category (*p*<0.0001). The vast majority of the tumors of both upper (83.6%) and lower (97.4%) lip were developmental/ reactive. In contrast, benign neoplasms accounted for a significantly higher percentage of the upper (14.1%) than the lower (1.8%) lip tumors. Seven malignant neoplasms involved the lower lip, accounting for 0.76% of lower lip tumors (Table 2, Fig. 1). Benign and malignant neoplasms had also significantly larger dimensions, compared to developmental/reactive tumors (*p*=0.001). Among the 1042 cases with available dimensions, 735 (70.5%) were up to 1 cm in maximum diameter; 97% of them were developmental/reactive tumors, 2.7% benign neoplasms and 0.3% malignancies (Table 2).

**Table 2:**
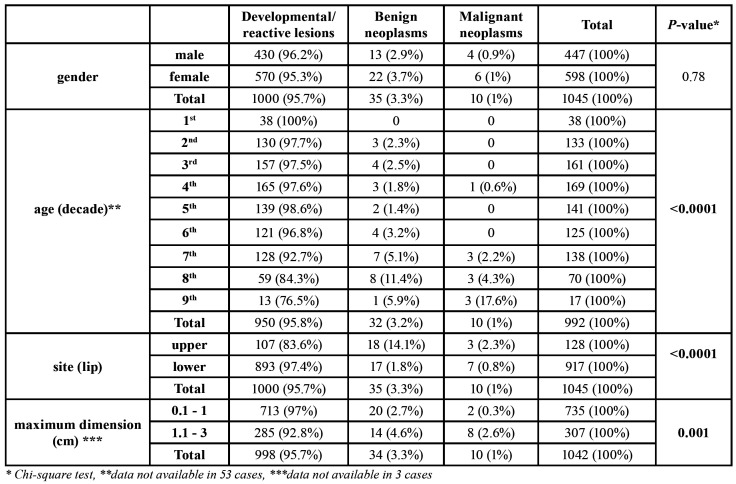
Distribution of 1045 localized lip swellings in diagnostic groups in asciation with gender, age, site and lesion’s maximum dimension.

Based on the aforementioned results, binary logistic regression was performed to estimate if gender, age equal or more than 60 years, lip tumor site and diameter above 1cm could serve as independent predictor variables for the occurrence of neoplasms (either benign or malignant). After adjustment for covariates, the analysis revealed that a lip tumor found in patients ≥60 years was 4.6 times more likely to be neoplastic compared to a lip tumor found in individuals younger than 60 years (*p*<0.0001); an upper lip tumor was 4.1 times more likely to be neoplastic compared to a lower lip tumor (*p*<0.0001); and a tumor of maximum diameter larger than 1cm was 34% more likely to be a neoplasm compared to tumors of diameter up to 1cm (*p*<0.0001). Gender was not found to be associated with the occurrence of neoplasm (*p*=0.249).

**Figure 2:**
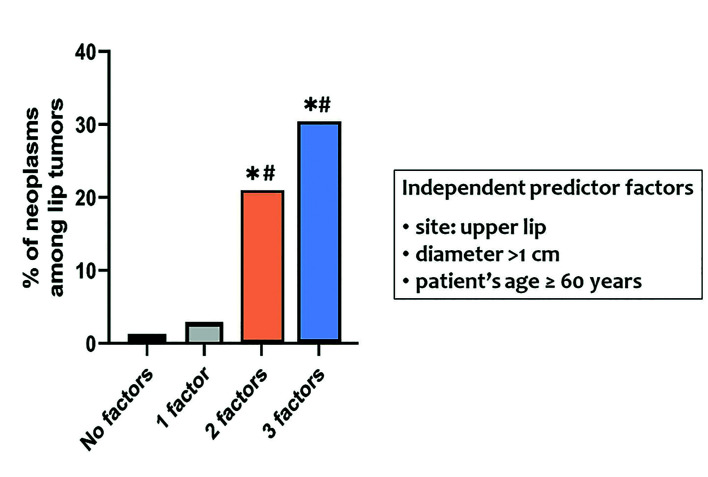
The probability for a lip tumor to be neoplastic was estimated based on the number of certain independent predictive factors, i.e. patient’s age equal or more than 60 years, tumor’s location in the upper lip and maximum diameter larger than 1cm. Lip tumors in patients who presented with 2 independent factors were 20.2 times more likely to be neoplastic compared to those presenting none of the above factors (<italic>p</italic> < 0.0001) CI (7.698, 53.109). Accordingly, patients with presence of all 3 predictive factors were 33.6 times more likely to have a lip tumor of neoplastic nature compared to those who presented with none of the factors (<italic>p</italic> < 0.0001) CI (9.637, 117.45) (CI = confidence interval). * indicates statistical difference compared to no factors. # indicates statistical difference compared to 1 factor.

Fig. 2 illustrates the probability for a lip tumor to be neoplastic based on the presence of certain independent predictive factors, i.e. patient’s age equal or more than 60 years, tumor’s location in the upper lip and maximum diameter larger than 1cm. Probabilities in this example are expressed as proportions (12), i.e. the probability of being a neoplasm when an independent factor is present is the proportion of neoplasms among all lip tumors in the sample population, when this independent factor is present. Lip tumors in patients who presented 2 independent factors were 20.2 times more likely to be neoplastic compared to those presenting none of them (p < 0.0001). Accordingly, patients with presence of all these 3 independent predictor variables were 33.6 times more likely to have a lip tumor of neoplastic nature compared to those who presented without them (p < 0.0001).

Twenty three (2.2%) cases had been submitted for histopathological examination without a clinical diagnosis, while a differential diagnosis was provided in 128 (12.2%) cases. As it is presented in Table 3 complete or partial agreement between the clinical and pathological diagnosis was seen in 96.3% of all cases.

Complete agreement (grade 0) was observed in 81.7% of developmental/reactive tumors, and disagreement (grade 2) was noticed in most benign (58.8%) and malignant (70%) neoplasms, resulting in a statistically significant association between the diagnostic group and grade of agreement (*p*<0.0001). Of grade 1 tumors, 10 out of 11 developmental/reactive tumors were clinically diagnosed as benign neoplasms and 1 as manifestation of amyloidosis; 19 out of 20 benign neoplasm were clinically misdiagnosed as developmental/reactive, while one case was considered as squamous cell carcinoma. In contrast, four malignant tumors (70%), i.e. 2 squamous cell carcinomas, 1 lymphoma and 1 cystadenocarcinoma, were excised with the provisional diagnosis of reactive tumors (pyogenic granuloma 2 cases, mucocele 1 case, chronic abscess 1 case), while 3 malignancies were clinically misdiagnosed as benign salivary gland tumors (2 cases) or benign mesenchymal tumor (1 case). Chi-square test did not reveal any significant association between the grades of agreement and the location of the tumors (*p*>0.05).

**Table 3:**
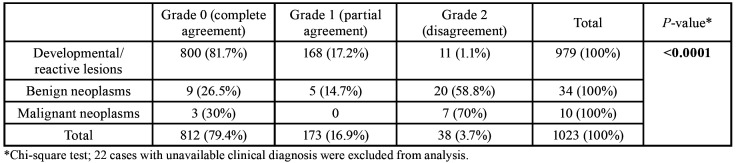
Grades of agreement between the clinical and the histopathological diagnosis in 1023 localized lip swellings.

## Discussion

To our knowledge the 1045 cases analyzed herein are the largest sample of lesions of the labial mucosa with histopathological confirmation of the diagnosis reported up to date. Previous retrospective studies included lip lesions diagnosed by clinical examination alone (4) or biopsy was performed in selected cases (5,13), as well as in both cutaneous and mucosal lesions (3,5,7,9,10) without a focus on localized mucosal swellings. Therefore comparison with our study is not possible.

The main findings of the present study support the clinical impression that although tumors of the lip mucosa arise more commonly in the lower lip and most of them are of developmental/reactive origin, tumors of the upper lip in patients ≥60 years old and/or of maximum diameter >1 cm have significantly higher probability to be of neoplastic origin.

Lip biopsies represented 12.9% of the total number of biopsies accessioned in a single oral pathology laboratory during a 10-year period, a percentage comparable to the 16.6% reported in a similar study from Brazil (9), and 74.3% of them were tumors. Although ethnical differences may exist, those findings highlight the high frequency of lip tumors among oral lesions (5,6) and emphasize the importance of clinicians’ knowledge about their diagnosis and management.

The main selection criteria for the cases included in the present study were the localization of the lesion on the lip mucosa and its description by the submitting clinician as a “tumor” in the preprinted biopsy request forms used in our laboratory. Considering the latter, although some of the lesions included do not fit the definition of a tumor as a well circumscribed, solid localized swelling, it reflects the clinicians’ impression. The diagnostic categorization of Allon *et al*. (11) that separates developmental/reactive tumors from neoplasms was followed, as it allowed addressing the main aim of our study, whether tumors of the upper lip are more commonly neoplasms compared to those of the lower lip. Benign vascular lesions, i.e. hemangiomas and vascular malformations, were included in the developmental/reactive group, as previously suggested (11).

In accordance with previous studies most lesions were located on the lower lip compared to the upper lip (3,7,9,10,13) and developmental/reactive tumors represented the largest category both for the upper and lower lip (3,9). The latter has been attributed to the greater exposure of the lower lip to factors, such as trauma or environmental insults (3,4,13). The most common lip tumor was mucous extravasation cyst, while other common diagnoses in this category were irritation fibroma, hemangioma and squamous papilloma, as in previous studies (3,5,9,13). Irritation fibroma, pyogenic granuloma and squamous papilloma were more common on the lower lip, but in other reports (9) the latter two are more common on the upper lip.

Mucous retention cysts showed a predilection for the lower lip of young patients, as in previous reports (7,9). Only seven mucous cysts were found on the upper lip and almost all of them were retention cysts, in contrast to the lower lip, where retention cysts represented approximately 1% of mucous cysts. Based on those findings, a tumor-like enlargement on the lower lip of a young patient is most probably a mucous extravasation cyst, while when a mucous cyst is included in the differential diagnosis of an upper lip tumor this is rather a retention cyst.

No gender predilection was documented among the diagnostic categories (3,7,9), while the mean age of male and female patients did not differ significantly. However, an age equal or higher than 60 years was an independent predictor for the diagnosis of neoplasms in the present study. Osterne *et al*. (9) reported reactive tumors to be more common among patients in 2nd to 5th decade of life, whereas malignancies involved patients with a mean age of 58 years. Α maximum diameter >1cm was also found to be associated with higher probability for a tumor to be a neoplasm. To the best of our knowledge, association between the size and diagnostic category of lip tumors has not previously been documented.

Considering benign neoplasms, lipomas and adenomas were the most common. The lips are the second most common site for oral and maxillofacial lipomas (14,15), although labial lipomas have been scarcely (9) or never (3-5,7,10,13) reported in other similar studies, and the lower lip is three times more commonly affected than the upper lip (14,15). The lips are, also, the second or third most common site for benign neoplasms of the minor salivary glands (16,17). Almost all (11/12) adenomas in the present study arose in the upper lip and most were pleomorphic adenomas, as documented by other authors (16,17). The number of benign neoplasms in both lips was almost equal, but due to the high number of developmental/reactive tumors in the lower lip, the relative frequency of benign neoplasms was 1.8% in the latter, in contrast to 14.1% in the upper lip.

Malignancies accounted for less than 1% of all lip tumors in the present study, involved mostly the lower lip, and were squamous cell carcinomas in older patients, mainly of 7th to 9th decade of life, as in previous reports (8,9,18). In a previous histopathological study of 995 lip lesions (9), 121 malignant tumors were found, but 114 of them involved the skin or the vermillion border of the lips.

Overall there was a high degree of agreement between the clinical and final diagnosis in the present study, although considerable differences in the level of experience of the submitting clinicians are expected due to its retrospective nature. Benign neoplasms accounted for most cases of disagreement, and this could be attributed to their rarity and clinical similarity with reactive lesions. Agreement for malignancies was found in 3 out of 10 tumors, when Curra *et al*. (19) reported a 97.4% success rate of clinical examination to identify potentially malignant and malignant lip lesions. As different kinds of lesions were included in the latter study, e.g. ulcers, plaques, tumors, direct comparison with our results is not valid. Clinical features of a lesion, such as color or consistency, are considered in the differential diagnosis, in addition to age, gender, tumor site and size. However, the former parameters require experience in Oral Medicine, in contrast to the latter that can be objectively evaluated even by non-specialists involved in the diagnosis and management of oral soft tissue lesions. It was this group of practitioners that we aimed to alarm on the possibility that a tumor of the upper lip may be a neoplasm.

The main limitations of the present study are that the material was retrospectively collected from a single oral pathology laboratory, and that the description of the lesions was based on the clinical impression of numerous submitting clinicians with different areas of specialization and years of experience.

In conclusion, the results of the present study confirmed our initial clinical observation that in contrast to lower lip tumors, which are commonly reactive, a considerable proportion of upper lip tumors are neoplasms, mostly benign. On the other hand, the malignant neoplasms are more likely to be found on the lower lip. Younger patients mainly present with developmental/reactive lip tumors that are usually up to 1 cm in diameter, while lip tumors measuring >1cm in patients ≥60 years have significant probability to be a neoplasm. Biopsy and histopathological examination is the gold standard for establishing the final diagnosis, as benign or even malignant neoplasms might be clinically misinterpreted as reactive lesions.
